# Reasons for encounter in young people consulting a family doctor in the French speaking part of Switzerland: a cross sectional study

**DOI:** 10.1186/s12875-015-0375-x

**Published:** 2015-10-30

**Authors:** Anne Meynard, Barbara Broers, Danièle Lefebvre, Françoise Narring, Dagmar M. Haller

**Affiliations:** Adolescent and young adult program, Department of Child and Adolescent Health, Geneva University Hospitals, 87 Bvd de la Cluse, 1205 Geneva, Switzerland; Dependencies unit, Division of primary care, Department of Community Medicine, Primary Care and Emergency, Geneva University Hospitals, 4 rue Gabrielle-Perret-Gentil, 1211, Geneva, 14 Switzerland; Private general practice 93 Route de Suisse, 1290 Versoix, Switzerland; Primary care research and teaching unit. Faculty of Medicine, University of Geneva CMU, 9 av de Champel, 1211, Geneva, Switzerland

**Keywords:** Young people, ICPC-2, Family medicine, Reason for encounter

## Abstract

**Background:**

Knowledge of patient’s reasons for encounter is useful to inform health service planning and health professional education. Our aim was to describe reasons for encounter as stated by an unselected group of young people attending primary care practices in the French-speaking part of Switzerland.

**Methods:**

Consecutive patients aged between 15 and 24 years were recruited as part of the PRISM-Ado trial (n = 594). They completed an anonymous questionnaire in the waiting room, including their main reason for encounter (free text). Reasons for encounter were coded using ICPC-2 classification and analyzed according to sex, age and living in a rural or urban area.

**Results:**

95 % of questionnaires contained valid data about reasons for encounter (n = 567). General and unspecific (A) reasons were the most common in boys (44 %) and girls (42 %), followed by respiratory, musculoskeletal, dermatological and psychological reasons. Psychological reasons were more frequent in girls attending urban practices; musculoskeletal and dermatological reasons were more frequent in rural areas. Sexually transmitted infections or substance use were very rarely stated as a reason for encounter.

**Conclusions:**

This is the first study describing reasons for encounter as stated by young people themselves in primary care in Switzerland. These findings provide useful guidance for family doctors training and health service planning in Europe.

**Trial registration:**

Australian New Zealand Clinical Trials Registry, ACTRN12608000432314.

## Background

Providing comprehensive care for the general population throughout the life course is the cornerstone of family medicine. Recent research emphasizes the importance of a lifecourse approach in adolescent health [1]. This includes for example addressing health compromising behaviours that usually start in adolescence and affect later life, ensuring immunization coverage, or encouraging a responsible use of health services. Family medicine represents the main point of access to care for young people in the world. In Switzerland as in other high income countries, most young people use primary care at least once a year with no differences between young people engaging in health compromising behaviours or not [2, 3].

Although adolescence is often seen as a period of opportunities and good health, the burden of disease in adolescents and young adults (10–24 years) is far from negligible. In all regions of the world there is an excess mortality in 15–24 year old boys mainly related to violent deaths (traffic accidents, violence and suicide). Girls’ mortality worldwide is still related to materno-fetal causes but in high income countries suicide and accidents are also the main cause of mortality in this age group [4]. In high income countries like Switzerland, mortality remains low but burden of disease as measured by DALY’s (disability adjusted life years) significantly rises from early to late adolescence especially around mental health, substance use but also iron deficiency and consequences of unsafe sex [5]. Evidence shows that public health campaigns and school health programs focusing on development of healthy environments and life skills among young people are effective [6].

Gaining a better understanding of young people’s reasons for encounter can help adapt training programs and sensitize family doctors to young people’s health needs. Primary care providers can reinforce public health messages at the individual level and encourage responsible use of health services with adolescents as they are their future adult patients. If adequately trained they can well respond to the needs of adolescents [7, 8].

The International Classification for Primary Care-2 (ICPC-2) is internationally recognized as the best tool for coding primary care medical encounters. The Swiss Society for General Medicine has therefore acknowledged ICPC as the official coding system for primary care and recently developed a project for better coding in primary care (FIRE project) [9–11]. FIRE provides a view of reasons for encounter from a doctors’ perspective and not from a patient’s point of view .It does not provide data on children or young people.

We reviewed three studies addressing self-reported health needs of young people in relation with family medicine, all of them using ICPC coding. In an Australian study describing the profile of 16–24 year old young people consulting a family doctor, 450 young people were interviewed confidentially and their reasons for encounter coded using ICPC-2 [12]. In Europe two studies focused on young people’s ICPC coded reasons for encounter. One was a German study (0–19 years old) the aim of which was to describe access to family medicine of children and adolescents aged 0–19 years old [13]. The other study was a Franco-Belgian study comparing reasons for encounter as reported by young people (12 to 17 years old) and those reported by family doctors for these same young people [14]. In all studies young people’s most frequent reasons for encounter with a family doctor were respiratory, general, musculoskeletal and skin reasons with some differences especially gynecological reasons being more frequent in the Australian study and very low in all the others. Substance use and mental health issues as well as injuries were rarely mentioned.

The rationale for our study was to focus on the period of transition between adolescence and young adulthood based on the World Health Organization definition of adolescence (10–24 years old). We were also interested to collect data on older adolescents and young adults as epidemiological data shows a rising burden of disease in these age groups.

To date, there are no published studies in Switzerland about young people’s utilization of primary care facilities: differences between urban and non-urban or geographical areas or reasons for encounter as described by young people themselves. The aim of our study was to describe young people’s stated reasons for encounter in private family medicine practices in Switzerland.

## Methods

### Study design and setting

This is a cross-sectional study conducted in family medicine practices (general internists and paediatricians) in the French-speaking part of Switzerland. Data were extracted from baseline data of a large primary care cluster randomised controlled trial (PRISM-Ado trial) conducted from February 2009 to November 2010 [15]. The PRISM-Ado trial tested a training intervention of family doctors to address substance use in adolescents.

### Swiss context of care

Switzerland has a universal health coverage system through private insurance schemes. As it is a federal state, every canton has different health regulations. There is easy access to primary care (paediatricians, general practitioners or general internists now all are members of a national association of family medicine). Family doctors do not necessarily act as gate-keepers, patients have free access to specialized care. Pediatricians are part of the primary care system and can follow their patients into young adulthood without restrictions although most paediatricians encourage transition to adult care around the age of 16.

The Swiss population especially in urban areas also has easy access to outpatient emergency clinics, hospital emergency units. Family planning clinics and gynecologists are the main source of care for sexual and reproductive health care. Recent data from the Swiss Health Observatory show that 60 % of the 15–34 year old see a family doctor at least once a year and 26 % a specialist. These data do not allow more refined analysis (whether they have seen both a family doctor and a specialist for example) [16].

According to cross-sectional population based data (The second Swiss Multicenter Adolescent Survey on Health) [17] three quarter of the adolescents has seen a family doctor at least once in that year, one third had seen a specialist and 50 % of girls had seen a gynecologist.

### Participants

Young people aged 15 to 24 years attending the practices of 32 physicians participating in the PRISM-ADO trial were included [15]. The choice of the age-range 15 to 24 years (“youth” according to the United Nations definition) was based on two considerations: young people in this age-group share a similar burden of disease throughout this developmental phase from adolescence into adulthood [18]. In addition, as from the age of 15 years they could participate in this low risk study without asking for parental consent [19]. The Swiss federal law on research states that parental consent is not required in low risk studies for adolescents less than 16 years old [20]. All consecutive patients in this age group attending the practice during the trial period were eligible. Patient exclusion criteria were: acute illness requiring immediate attention of the physician, severe mental disorder such as psychosis or suicidal thoughts, drug or alcohol abuse requiring more immediate attention (i.e. alcohol abuse and recent court ruling regarding drunk driving), previous treatment for cannabis or alcohol dependence, inability to read and understand French, any other disorder affecting the capacity of the young person to consent on their own to participate in the study. Participating practices were not chosen at random: family doctors volunteered to participate in the trial. However the sample was varied with representatives of different age groups and both genders, single, double or group practices, in rural or urban settings (Table 1). Acceptance rate of participation by the patients was high (594/666 = 89 %), so we can consider our study population as an unselected group of young adults consulting their primary care physician. 32 patients were excluded because of an acute illness, 40 patients denied participation. Details about acute illness leading to exclusion and reasons for refusals were not systematically recorded.

**Table 1:** Characteristics of the 32 participating family doctors

Characteristics	N (%)	National data 2012^a^ (%)
*Socio-demographic characteristics*		
Age group,		
36 to 45 years old	15 (47)	(19)
46 to 55 years old	12 (37)	(36)
>55 years old	5 (16)	(44)
Male	18 (56)	(70)
*Professional characteristics*		
Specialty = paediatrics (as opposed to general internal medicine)	5 (16)	NA
Solo or duo practice	25 (78)	(85)
Practice located in urban area	22 (69)	NA

^a^The Commonwealth Fund, International Health Policy Survey 2012 [22]

### Instruments and procedure

The practice assistant recruited eligible patients as they came in for their consultation. Prior to the consultation, participants were invited to complete a confidential survey with questions evaluating general health, substance use, socio-demographic and psychosocial variables. They completed the survey in the waiting room, or if this did not provide sufficient privacy, in a separate room. The young people always completed the questionnaire before the medical encounter and were asked for practical reasons (time constraint associated with research in a busy primary care practice) to complete only the main reasons for encounter::

“*Finally, thank you for stating with your own words the main reason for you to come today (for example: common cold, school problem, prescription for contraceptives, sadness, ankle sprain,…)?*”

Patients completed it in free text and the investigators then coded these complaints using the **I**nternational **C**lassification for **P**rimary **C**are 2 (ICPC-2).

ICPC-2 classification (www.icpc.ch)

The structure of ICPC-2 classification is organized in the following way: 17 main chapters from A to Z and for each chapter 7 identical components (eg: symptoms/complaints, infections, neoplasm, injuries, congenital anomalies, other diagnosis). It also contains a section on procedures (blood test, immunization,..). The investigators were familiar with this classification as they had used it in previous studies [12, 21]. The young people filled in free text before the medical consultation. The questionnaire was then anonymously collected and therefore not seen by the doctor or practice assistant. A research assistant (medical student not involved in the care of the patients) coded all the patients’ answers using ICPC-2 codes. Procedural codes were related to the corresponding chapter. Ambiguous answers were discussed and consensus sought with one of the main investigators (DMH) to improve reliability of the coding strategy.

Examples: “ I come because I feel down” or “ I come because I feel sad” was coded P03 “Sensation of depression”

“I come to get a medical certificate for vocational school” was coded A98 and I come to get an immunization” was coded (code procedures) A44”

### Analysis

Data were entered using Epidata and a descriptive analysis of the ICPC-2 coded reasons for encounter was undertaken using Stata software.

Reasons for encounter were analyzed by frequency of occurrence. Analysis was done by chapter (ICPC-2 list A to Z) and by individual reason for encounter and then analyzed by age group, gender as well as by geographical distribution of the practices (urban, if >15’000 inhabitants, or non urban areas). Two age groups were considered: 15 to 19 and 20 to 24 years old. The association between reasons for encounter and age groups, gender and practice location was examined using multivariate logistic regression adjusting for these variables and clustering within practices.

The PRISM-Ado study protocol was approved by the Ethics Committee for Studies in Outpatient Care, Geneva University Hospitals, Geneva Switzerland (protocol 08–28). Ethical approval for the PRISM-Ado trial included approval of baseline data collection and descriptive analysis. The baseline questionnaire, including questions about reasons for encounter was submitted and approved by the ethical committee.

## Results

### Sociodemographic data

594 young people completed the study questionnaire. The mean age was 18.5 (±2.6) years old, 53 % were female. 55 % of the study population were students, mostly living in urban areas (61 %), more than 80 % were born in Switzerland.

### Reasons for encounter

Of 594 questionnaires completed for the PRISM-Ado trial, 567 (95 %) had an answer to the question of reason for encounter that could be coded. The most frequently cited ICPC-2 chapters were A (general + procedural reasons for encounter all related to chapter A) 43 %, R (respiratory) 16 % and L (musculoskeletal) 12 %.

**Fig. 1:**
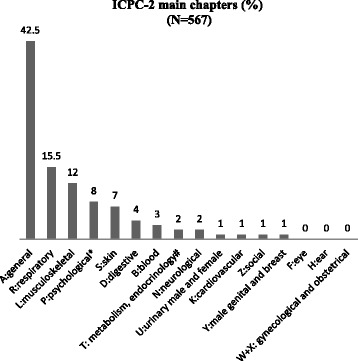
ICPC-2 classification of reasons for encounter (percentages only). * Psychological includes substance use and eating disorders # Metabolism/endocrinology includes weight gain, obesity ICPC-2 (International Classification for Primary Care-2) is divided in 17 main chapters from A to Z classified by organs and systems, except chapter Z representing social codes. Procedures are included in the A chapter. In this sample the most frequently cited chapters are A: General, R: Respiratory, L: Musculoskeletal. All chapters were cited at least once except F: Eye, H: Ear W: Pregnancy, Childbearing, Family Planning and X: Female Genital.

Sensitive topics such as substance use, sexual and reproductive health motives were almost not expressed by young people as reasons for encounter Fig. 1.

### Reasons for encounter according to age, gender and practice location

Table 2 lists all the reasons for encounter and Table 3 illustrates the top ten reasons for encounter in males and females and according to the location of the practice (urban or non-urban). General check up was the most frequent reason both in males and females (43.2 % /41.9 %), younger or older age groups and urban or non-urban practices. Immunization represented 7.2 % of reasons stated. Musculoskeletal and respiratory reasons were more frequent among males and in non-urban practices but these differences were not statistically significant. Blood (B) reasons for encounter were significantly more frequent among girls. There were no statistically significant differences between the younger and the older age group for all reasons for encounter. Psychological and digestive symptoms were statistically significantly more frequent, skin complaints statistically significantly less frequent in urban practices.

**Table 2:** ICPC-2 coded main categories of reasons for consulting as indicated by young people before their consultation and most frequent subcategories (total frequency > 3), by gender, by age-group and by practice location (numbers are percentages)

Reason for encounter (main ICPC categories)	Males (N = 259)	Females (N = 308)	15-19 years (N = 307)	20-24 years (N = 260)	Visiting in urban practice (N = 353)	Visiting in non-urban practice (N = 214)	All participants (N = 267)	Measures of association^a^
A:general	43.0	41.8	43.0	41.9	44.8	38.9	42.5	no significant difference
*Health maintenance/prevention* A98	20.8	18.2	23.2	11.8	19.3	19.6	19.4	
*Preventive -immunisation/Medication* −44 (A44)	6.9	7.5	6.6	8.6	7.9	6.1	7.2	
*General symptom/complaint other* A29	4.6	5.5	5.5	4.3	5.4	4.7	5.1	
*Weakness/tiredness general* A04	0.4	2.9	1.6	2.1	2.3	0.9	1.8	
*Blood test −*34(A34)	1.2	1.6	1.0	2.1	2.0	0.5	1.4	
*Results exam/test/record* -61(A61)	1.2	1.6	1.3	1.6	1.4	1.4	1.4	
*Allergy/allergic reaction NOS* A92	1.9	1.0	1.0	2.1	2.0	0.5	1.4	
*Trauma/injury NOS* A80	2.7	0.3	0.8	2.7	1.1	1.9	1.4	
*General disease NOS* A99	1.5	1.0	1.0	1.6	0.8	1.9	1.2	
R:respiratory	17.8	13.6	15.6	15.4	12.7	20.1	15.5	no significant difference
*Upper respiratory infection acute* R74	8.9	4.2	6.8	5.4	4.5	9.3	6.4	
*Tonsilitis acute* R76	3.3	2.3	2.6	3.2	2.5	3.3	2.8	
*Cough* R05	1.5	1.9	2.1	1.6	1.1	2.8	1.8	
*Sneezing/nasal congestion* R07	0.8	2.3	2.7	1.0	1.4	1.9	1.6	
L:musculoskeletal	14.3	10.1	12.4	11.5	9.6	15.9	12.0	no significant difference
*Sprain/strain of ankle* L77	3.1	1.3	2.6	1.1	2.0	2.3	2.1	
*Back symptom/complaint* L02	2.3	1.3	2.4	0.5	1.7	1.9	1.8	
*Knee symptom/complaint* L15	0.8	1.9	1.3	1.6	2.0	0.5	1.4	
*Hand/finger symptom complaint* L12	0.8	1.6	1.3	1.1	0.6	2.3	1.2	
*Foot/toe symptom complaint* L17	1.9	0.3	1.3	0.5	0.3	2.3	1.1	
*Sprain/strain of joint NOS* L79	0.8	1.3	1.0	1.1	0.8	1.4	1.1	
S:skin	8.1	6.8	7.8	6.9	4.5	12.2	7.4	significantly less frequent in urban compared to non-urban locations^b^
*Acne* S96	3.5	1.0	3.2	0.0	1.4	3.3	2.1	
*Skin symptom/complaint other* S29	1.5	1.6	1.3	2.1	0.8	2.8	1.6	
*Warts* S03	0.8	1.0	1.3	0.0	0.6	1.4	0.9	
P:psychological	4.3	8.8	6.2	7.3	8.9	3.3	6.7	significantly more frequent in urban compared to non-urban locations^c^
*Feeling depressed* P03	1.5	3.9	2.1	4.3	3.7	1.4	2.8	
*Anorexia nervosa/bulimia* P86	0.0	2.3	1.6	0.5	2.0	0.0	1.2	
*Feeling anxious/nervous/tense* P01	1.2	1.0	0.8	1.6	1.4	0.5	1.1	
*Depressive disorder* P76	0.4	1.0	0.5	1.1	0.8	0.5	0.7	
D:digestive	2.7	5.2	3.3	5.0	5.9	0.9	4.1	significantly more frequent in urban compared to non-urban locations^d^
*Abdominal pain/cramps general* D01	0.0	1.9	0.8	1.1	1.4	0.0	1.1	
*Digestive symptom/complaint other* D29	1.5	0.6	0.8	1.6	1.4	0.5	1.1	
*Heartburn* D03	0.0	1.3	0.5	1.1	1.1	0.0	0.9	
B:blood	0.5	4.5	2.9	2.3	2.5	2.8	2.7	significantly more frequent in females compared to males^e^
*Aneamia other/unspecified* B82	0.0	1.9	1.3	0.5	1.4	0.5	1.1	
*Iron deficiency anaemia* B80 *(or blood test for iron, B34)*	0.0	1.3	0.8	0.5	0.6	0.9	0.7	
T: metabolism, endocrinology	1.9	1.9	1.6	2.3	2.3	1.4	1.9	no significant difference
*Endocrine/met/sympt/complt other* T29	1.2	0.6	0.3	2.1	1.1	0.5	0.9	
N:neurological	1.9	1.6	2.3	1.2	2.3	0.9	1.8	no significant difference
*Headache* N01	1.2	1.0	1.0	1.1	1.4	0.5	1.1	
Z: Social problems	1.5	0.3	1.3	0.0	1.1	0.5	0.9	no significant difference
*Social problems NOS* Z29	1.2	0.3	1.0	0.0	0.8	0.5	0.7	

^a^Logistic regression adjusted for age-group, gender, urban location and clustering within practices

^b^OR = 0.35; 95 % CI = 0.12-0.98; p = 0.047

^c^OR = 2.7; 95 % CI = 1.3-5.5; p = 0.007

^d^OR = 6.5; 95 % CI = 1.5-28.0; p = 0.013

^e^OR = 13.4; 95 % CI = 1.6-114.1; p = 0.019

**Table 3:** Top Ten Reasons for encounter by gender and location of practice

	Top ten reasons for encounter					
	Males	Females	Urban practices		Non-urban practices	
1	Health maintenance/prevention	Health maintenance/prevention	Health maintenance/prevention		Health maintenance/prevention	
2	Upper respiratory infection acute	Preventive immunisation/Medication	Preventive immunisation /Medication		Upper respiratory infection acute	
3	Preventive immunisation/Medication	General symptom/complaint other	General symptom / complaint other		Preventive immunisation /Medication	
4	General symptom/complaint other	Upper respiratory infection acute	Upper respiratory infection acute		General symptom/complaint other	
5	Acne	Feeling depressed	Feeling depressed		Tonsilitis acute	
6	Tonsilitis acute	Weakness/tiredness general	Tonsilitis acute		Acne	
7	Sprain/strain of ankle	Tonsilitis acute	Weakness/tiredness general		Cough	
8	Trauma/injury NOS	Sneezing/nasal congestion	Blood test	Allergy/allergic reaction NOS	Skin symptom/complaint other	Sprain/strain of ankle
9	Back symptom/complaint	Anorexia nervosa/bulimia	Sprain/strain of ankle_Knee symptom	Anorexia nervosa/ bulimia	Hand/finger symptom complaint	Foot/toe symptom complaint
10	Allergy/allergic reaction NOS	Cough				

## Discussion

This is the first Swiss study looking at reasons for encounter in primary care for young people.

The most common reasons for encounter cited by participants in this sample are visits for general motives (such as health maintenance or immunization) accounting for more than 40 % of visits with no difference between genders or age groups. Respiratory and musculoskeletal complaints are also frequent in all subgroups. Young people consulting in an urban practice have more psychological and digestive complaints and young people consulting in a non-urban practice have more dermatological and musculoskeletal complaints.

Our results add to the two previous European studies showing data for older adolescents and young adults and from the patient’s perspective in another European region. Interestingly our findings are quite comparable to those of the Franco-Belgian study of adolescents aged 12 to 17 years, despite the age difference [12].

As stated earlier, the SMASH survey indicated that 50 % of girls had seen a gynecologist in the current year [17]. We hypothesize that this is still the case in the western region of Switzerland where the study was conducted and thus could explain the low number of gynecological reasons for encounter in our study (as in the Franco-Belgian study). In the Australian study gynecological reasons for encounters were among the most frequent reasons for encounter for girls suggesting differences in health care systems and the role of family doctors.

Substance use was hardly mentioned by young people themselves despite the fact that many of them were excessive users of cannabis, alcohol or tobacco. [15] Young people might not see these as a medical problem or might not want to raise the topic. Substance use was also a rare reason for encounter in previous studies.

Interestingly, ten years ago in a study of health professionals’ adolescent health training needs in Switzerland, the need for more training opportunities in management of complex situations (mental health, functional disorders in particular) stood out as an important need [5]   Our current findings add to this by indicating the need to appropriately train family doctors to deal with common complaints such as respiratory and musculoskeletal complaints in adolescents as well as with vague complaints such as dizziness and unspecific pain for example. Postgraduate training and continuous medical education in adolescent health for family doctors should include a strong focus on adolescent preventive care, mental health and effective communication skills.

### Strengths and limitations

ICPC coding was used based on the patients’ words and not the doctors’ interpretation of patient words as is often the case. Data was collected in a confidential context among consecutive patients consulting a primary care practitioner. ICPC coding was done rigorously with an experienced coder checking all codes.

Our study also had limitations. The sample of health professionals was not random, and involved family doctors with a special interest in research, in young people and in quality of care. Overall, in our study, family doctors were younger and often female compared to the population of family doctors in Switzerland. However, the sample was varied with representatives of different age groups and both genders, single, double or group practices, in rural or urban settings, as shown in Table 1. Our hypothesis is that often older doctors no longer take on new patients. Therefore adolescents and young adults who seek a new family doctor turn to younger doctors. Thus it is not surprising that this younger age-group is overrepresented in our sample of family doctors volunteering for a study on the health of young people attending primary care [22]. Reassuring is that data from the Swiss Sentinel Surveillance Network [23] show a gender balance of 47.6 % of boys and 52.4 % of girls in the 15 to 24 year old age group of patients consulting family doctors in Switzerland. The gender balance in our study population is very similar (47 % boys and 53 % girls).

Since all consecutive patients were included, selection bias at the patient level was also limited. As there was no data collection on reasons for refusals it is not possible to know if these patients suffered from more severe condition or substance abuse problems, yet the proportion of patients who declined participation was low. Another limitation was that young people could only state one reason for encounter and therefore some motives might have been missed. In the Franco-Belgian study the majority of patients stated only one reason for encounter, in the German study the mean number of reasons for encounter/patient was 1.5.

### Implication for practice and research

Preventive care is an important part of the work of a family doctor but nowadays mainly focusing on the needs of aging population with complex chronic problems. Social workers and school professionals (nurse, psychologists, teachers,..) are crucial partners to develop effective interventions for young people, especially the most vulnerable ones [17]. Population based data and school health surveys show intentional and unintentional injuries as well as mental health issues to be the main burden of disease in young people in high income countries [4]. Recurrent pain (headache, stomachache or musculoskeletal) or vague complaints such as fatigue are often linked with mental health issues (anxiety, poor life satisfaction,..) [24].

The high frequency of general or preventive reasons for encounter as well as vague complaints or musculoskeletal reasons for encounter in our study as stated by young people themselves highlights the preventive role family doctors play in young people’s health. These findings have to be integrated in pre and postgraduate training as well as in continuous education for medical doctors and other health professionals. Too often training sessions in relation to young people’s health focus on sexual health or substance use and therefore do not respond to young people’s or family doctors expressed needs.

Future work could compare, as done in the Franco-Belgian study, patient and health care providers’ views using ICPC coded reasons for encounter, in order to assess the extent to which data from the medical file (such as those collected in the FIRE project) can similarly be used to assess patients’ needs [9]. It could also be interesting to compare our results to reasons for encounter at different entry points such as emergency outpatient clinics which are known to attract more vulnerable populations with no regular source of care. Further research could also explore which topics are truly addressed during the medical encounter, regardless of the presenting reason. This could provide useful information as to the extent to which family doctors respond to the current health needs of adolescents and young adults during routine consultations.

## Conclusions

Our findings provide useful guidance for family doctors training and health service planning in Europe. They underline the importance of research in private practices to improve health care and prevention in youth as well as linking population based and school health surveys data to clinical care research. Effective interventions to decrease burden of disease, especially mental health and substance use need to address environmental, family and individual factors .As for other age groups family doctors play a central role if effectively trained to address individual needs of patients as well as being able to collaborate in an active network.

## Abbreviations

ICPC-2: International Classification for Primary Care-2
